# Gene Therapy Induces Antigen-Specific Tolerance in Experimental Collagen-Induced Arthritis

**DOI:** 10.1371/journal.pone.0154630

**Published:** 2016-05-09

**Authors:** Sara Tengvall, Tove Eneljung, Pernilla Jirholt, Olof Turesson, Kajsa Wing, Rikard Holmdahl, Jan Kihlberg, Anna Stern, Inga-Lill Mårtensson, Louise Henningsson, Kenth Gustafsson, Inger Gjertsson

**Affiliations:** 1 Department of Rheumatology and Inflammation Research, University of Gothenburg, Gothenburg, Sweden; 2 Medical Inflammation Research, Dept of medical Biochemistry and biophysics, Karolinska Institutet, Stockholm, Sweden; 3 Southern Medical University, Guangzhou, PR China; 4 Department of Chemistry, BMC, Uppsala University, Uppsala, Sweden; 5 Molecular and Cellular Immunology Section, UCL Institute of Child Health, London, United Kingdom; 6 Sahlgrenska University Hospital, Gothenburg, Sweden; Penn State University, UNITED STATES

## Abstract

Here, we investigate induction of immunological tolerance by lentiviral based gene therapy in a mouse model of rheumatoid arthritis, collagen II-induced arthritis (CIA). Targeting the expression of the collagen type II (CII) to antigen presenting cells (APCs) induced antigen-specific tolerance, where only 5% of the mice developed arthritis as compared with 95% of the control mice. In the CII-tolerized mice, the proportion of Tregs as well as mRNA expression of SOCS1 (suppressors of cytokine signaling 1) increased at day 3 after CII immunization. Transfer of B cells or non-B cell APC, as well as T cells, from tolerized to naïve mice all mediated a certain degree of tolerance. Thus, sustainable tolerance is established very early during the course of arthritis and is mediated by both B and non-B cells as APCs. This novel approach for inducing tolerance to disease specific antigens can be used for studying tolerance mechanisms, not only in CIA but also in other autoimmune diseases.

## Introduction

The main feature of rheumatoid arthritis (RA) is loss of tolerance to self-antigens followed by inflammation and joint destruction. Immunosuppressive drugs reduce the immune response to self-antigens but unfortunately they also increase the risk for infections [1]. An ideal way to treat RA would be to re-establish tolerance without a general suppression of the immune system. However, therapeutic tolerance has not yet been achieved in clinical trials [2], largely because the tolerogenic mechanisms are poorly understood.

The most commonly used mouse model for RA is collagen type II (CII)-induced arthritis (CIA). CII-specific tolerance in CIA depends on presentation of an already identified CII-peptide (amino acids (aa) 259–270) [3] in complex with the major histocompatibility complex type II (MHCII) Aq molecule [4,5]. To study tolerance mechanisms, the CII protein or CII-peptides in their native or modified forms have been administered via various routes and at different time points before induction or during the course of CIA: e.g. orally [6–8] intravenously [9], intraperitoneally [10] or nasally [11,12]. It has become apparent that the glycosylation pattern of CII is of major importance for tolerance induction in CIA [13,14], and that also truncated forms of the CII-peptide [15] can induce tolerance in mice provided that they still bind to the Aq molecule. These and other studies have provided invaluable data, but they have also highlighted at least two complications in tolerance induction. First, the tolerogen can also trigger a pathogenic immune activation under circumstances when the tolerogen has adjuvant properties, a risk that is increased if proteins are administered exogenously [16]. Second, the half life of the tolerogen is too short to be effective, e.g. when administered as peptides [17], and thereby only presented to the immune system at delivery time points. However, the mechanisms maintaining tolerance vary during the course of CIA and thus, even if tolerization time points prior CII-immunization are fine-tuned, a system that targets antigen presenting cells (APCs) and allows continuous expression of a tolerogenic CII-epitope is lacking.

Tolerance is not due to lack of immune stimulation but rather an active immune regulation that involves several different cell types. Adoptive transfer of CII-pulsed dendritic cells (DCs) [18,19] or indoleamine 2,3-dioxygenase (IDO) expressing [20,21] DCs from either orally CII tolerized mice or *in vitro* manipulated DCs have been found to alleviate, but not abrogate arthritis development. The effect seemed mainly to be attributed to induced Foxp3^+^ regulatory T cells (Tregs). Further supporting a role for Tregs in tolerance to CIA, another study showed a significant increase in the number of Tregs in CII tolerized mice [22]. B cells and CII-specific autoantibodies are hallmarks in CIA, and in the great majority of tolerance studies CII-specific antibodies decrease. However, not only the CII-specific antibodies, but also the B cells themselves are important in CIA, as B cell depletion delays the onset of arthritis [23]. B cells also seem to take an active part in tolerance induction since presentation of self-antigen by B cells results in Treg induction rather than in anergy or clonal deletion [24]. Also B regulatory cells are of importance for regulation of the joint specific immune response and development of CIA [25–28].

The suppressors of cytokine signaling (SOCS) protein family, which are induced by various cytokines, e.g. IL-10 [29] are important regulators of immune responses. The SOCS proteins exert their functions through negative feedback on the JAK/STAT pathway [30,31]. SOCS have been strongly implicated in regulation of autoimmune conditions, e.g. enhanced SOCS1 expression in NZB/W mice correlated with reduced IFN- ɣ production and restored the IFN-ɣ signaling pathway [32]. Further, up-regulation of SOCS1 and/or 3 in CIA coincided with increased IL-10 production and reduced severity of arthritis [33,34]. These effects are partly due to the importance of SOCS proteins in sustaining the regulatory phenotype of FoxP3^+^ cells and to create a tolerogenic cytokine milieu that prevents autoimmune reactions [35,36].

We have previously shown an induction of partial tolerance in CIA upon intravenous injection of lentiviral particles that code for the expression of the CII-peptide aa259-270 on MHCII in a fraction of host cells [37,38]. We hypothesize that a further increased expression of the tolerogen would induce complete CII-specific tolerance in CIA and allow detailed studies of immunological mechanisms. To this end we developed a mouse model using lentiviral based gene therapy in which the tolerogen is permanently expressed on MHCII Aq already before CIA is induced. In this model an almost complete and sustainable tolerance to CIA is established very early after immunization and shows the immunological complexity of tolerance and allows for further mechanistic studies in the field.

## Material and Methods

### Lentiviral constructs

**Fig 1:**
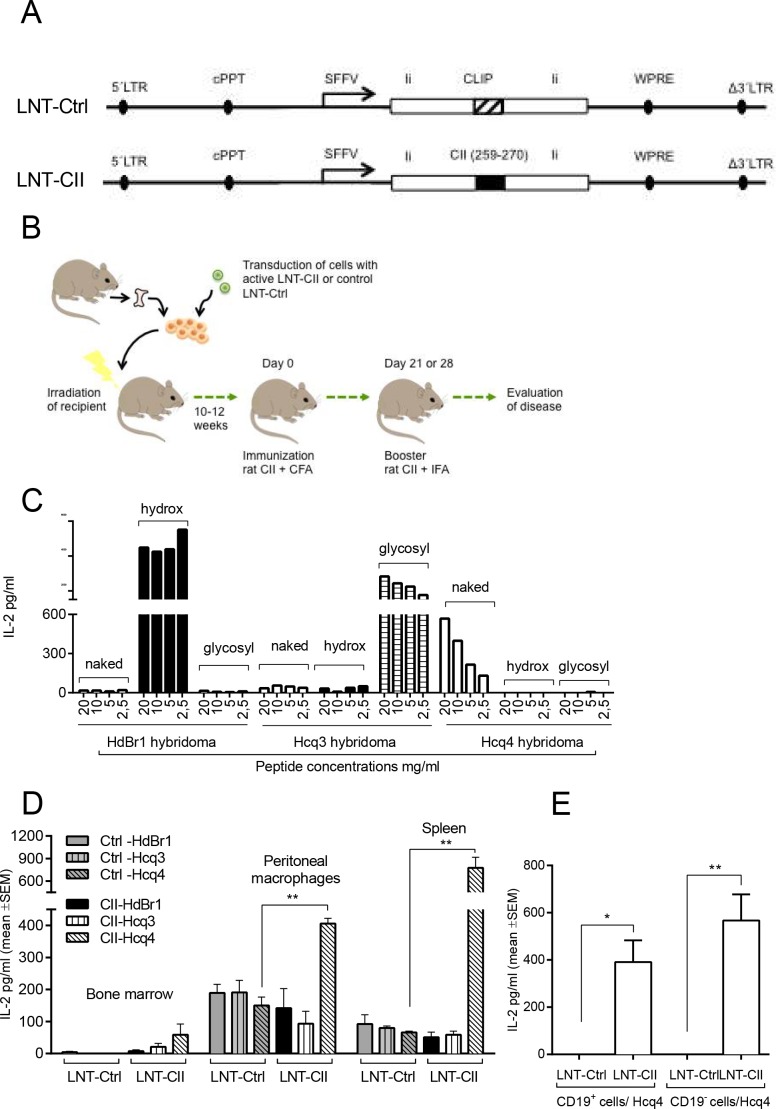
**Lentiviral constructs and model design (A)** LNT-Ctrl; lentiviral control construct that encodes the MHC class II–associated invariant chain peptide (CLIP) stabilizing Invariant chain (Ii) driven by the spleen focus-forming virus (SFFV) promoter. LNT-CII; lentiviral construct that encodes the rat collagen epitope (aa259-270, GIAGFKGEQGPK) fused into Ii. LTR; long term repeat, cPPT; central polypurine tract, WPRE; Woodchuck Post Transcriptional Element. **(B)** Cartoon describing the design of the experiment. Hematopoietic stem cells (HSCs) from DBA/1 mice were transduced with lentiviral particles injected into lethally irradiated recipient mice. CIA was induced according to standard protocol at least 10 weeks after HSC transplantation. **(C)** Naïve splenocytes were stimulated with naked, hydroxylated or glycosylated peptides and co-cultured with T cell hybridomas specific to each post-translational modification of the CII-peptide, i.e. Hcq4 recognizes the naked peptide, HdBr1 the hydroxylated and Hcq3 the glycosylated CII-peptide. The response was measured as IL-2 production by ELISA and performed three times. **(D)** IL-2 production in response to co-culture of the T cell hybridomas with bone marrow cells, peritoneal macrophages or spleen cells from HSC transplanted mice (n = 6/group) and performed twice. **(E)** Co-cultures of the T cell hybridomas with B cells (CD19^+^) or non-B cells (CD19^-^) from LNT-Ctrl and LNT-CII HSC transplanted mice (n = 3/group) and performed twice. Statistical analysis was performed by Student’s t-test, mean±SEM.

Generation of the CII expressing lentiviral vector (LNT-CII) has been described previously [37] (Fig 1A). Briefly, CII aa259–270 or CLIP amino acids were cloned into the class II–associated invariant chain peptide position of the invariant chain (Ii) to achieve efficient loading of and binding to MHCII. Next, the generated Ii-fragments were subcloned into the lentiviral vector pHR’SIN-cPPT-SFFV and named; LNT-CII and LNT-Ctrl. SFFV was used as a general promoter that ensures a high expression in all cell types.

### Production of lentiviral particles

Vesicular stomatitis virus—G pseudotyped lentivirus was produced by transient transfection of 293FT cells with three plasmids; the self inactivating transfer vector plasmid LNT-CII or LNT-Ctrl, the multi-deleted packaging plasmid; pCMVΔR8.74 and the VSV-G envelope; pMD.G2 and titrated as previously described [38]

### Mice

Male DBA/1 mice 6–8 weeks old were obtained from Taconic (Europe A/S, Ry, Denmark) and housed in a pathogen-free barrier facility (12-hr light/12-hr dark cycle) and fed water and rodent chow. The chow was placed on the cage floor and the water bottle tip was reachable when the mice stood on four paws. All mice were monitored daily. All animal studies were approved by the local Animal Ethics Committee in Gothenburg, i.e. Göteborgs Djurförsöksetiska Nämnd, Kammarrätten i Göteborg, Box 1531, SE401 50 Göteborg, Sweden according to Swedish legislation. Ethical approvements and approvement dates: 277–2011 (2011-09-11) and 233–2014 (2014-12-17). According to the ethical permission the mice were sacrificed by injection of an intraperitoneal injection of ketamine/medetomidin followed by opening of thorax and heart puncture. If the mice were affected by arthritis in the forepaws, scored higher than 5, they were euthanized as described above, and culled. In the present experiments, no mouse reached a score higher than 5 in the forepaws.

### Lentiviral transduction and transplantation hematopoietic stem cells

Femurs from DBA/1 mice (Taconic) 7–10 weeks of age were obtained and the femurs were clean of muscles and tendons and subsequently hematopoietic stem cells were purified using Mouse Hematopoietic Progenitor Cell Enrichment Kit (Stemcell Technologies, Manchester, UK). Purified hematopoietic stem cells were cultivated at a concentration of 1x10^6^ cells/ml in a 12-well plate in StemSpan (Stemcell Technologies) overnight in the presence of 100ng/ml mSCF, 100ng/ml Flt-3L, 100ng/ml IL-11, 20ng/ml IL-3 (R&D Systems, Abingdon, UK) and lentiviral particles at multiplicity of infection 75 (i.e. 75 lentiviral particles/cell). The cells were resuspended in PBS the following day and subsequently injected intravenously (2.5x10^5^ cells/mouse) into syngenic lethally irradiated (8.5 Gray) recipient mice. The cells were allowed to repopulate the mice for a minimum of 10 weeks before start of the experiments (Fig 1B).

### Collagen-induced arthritis

**Fig 2:**
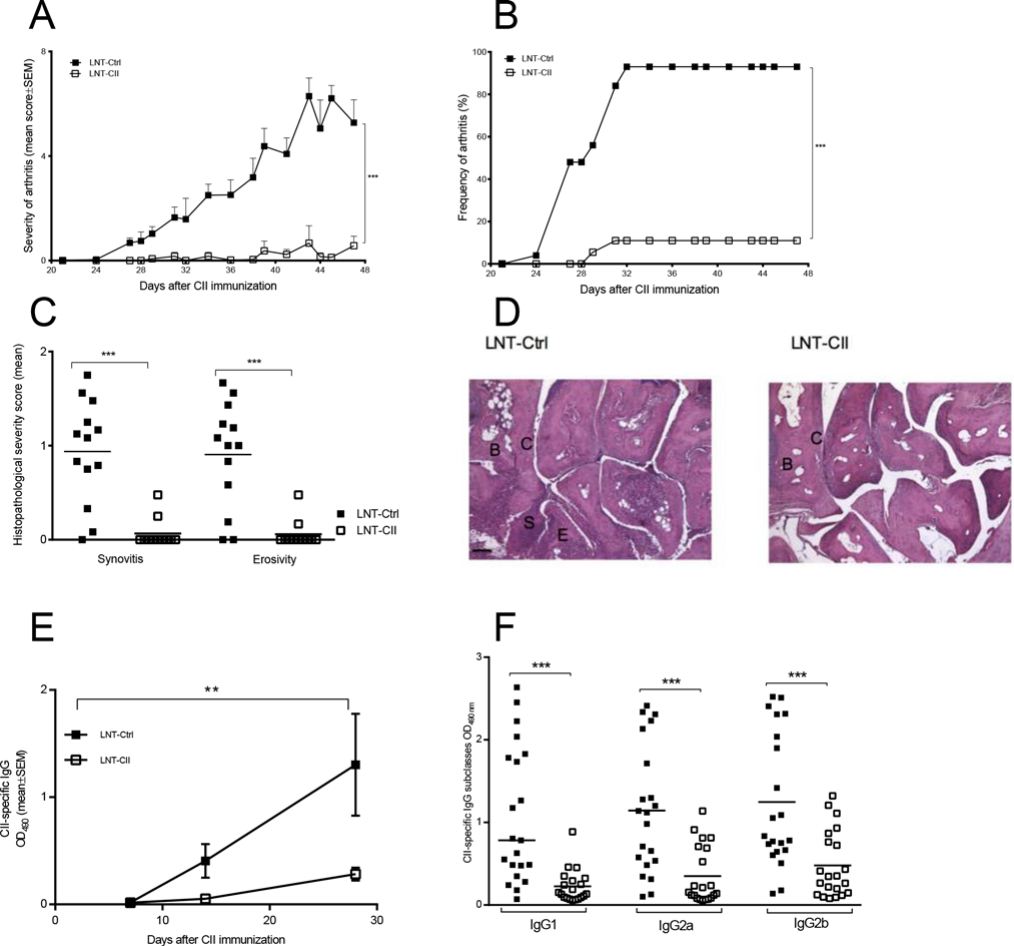
Development of arthritis and B cell responses in the novel model of tolerance to collagen-induced arthritis. Severity **(A)** as well as incidence of arthritis **(B)** were determined by macroscopical examination (LNT-Ctrl n = 25 (day 0–42), n = 17 (day 43–49) LNT-CII n = 24 (day 0–42), n = 11 (day 43–49)). The experiments have been repeated five times and the graph shows the pooled results from three experiments **(C)** Histopathological examination of synovitis and bone/cartilage erosivity at termination of two pooled experiments (days 42–49). **(D)** Histological photos from an LNT-Ctrl and an LNT-CII mouse at day 42 after CII immunization, B = bone, C = cartilage, S = synovium. Scale bar 100 μm. **(E)** Development of CII specific IgG antibodies measured by ELISA in serum at indicated time points during CIA (n = 3-6/group). **(F)** Serum levels of CII-specific IgG and subclasses IgG1, IgG2a and IgG2b at days 42–49. Experiments in Fig E and F have been performed at least twice. Statistical analysis was performed by linear regression in (A, D), logistic regression in (B) and Mann-Whitney U-test in (C and E), mean±SEM.

The mice were immunized with rat CII and complete Freund’s adjuvant (CFA, Sigma-Aldrich, Sweden) and boosted at day 21 or 28 (according to standard collagen-induced arthritis protocol [37]). Day of booster was changed from day 21 to 28 in the experiment shown in Fig 2D due to changes in the Swedish legislation. Arthritis severity and frequency were graded blindly and the arthritis (defined as visible erythema and/or joint swelling) was evaluated by inspection of finger/toe and ankle/wrist joints. A score was assigned for each limb: 0, neither swelling nor erythema; 1, mild swelling and/or erythema; 2, moderate swelling and erythema; and 3, marked swelling and erythema. The total score for each mouse was calculated by adding up the scores for each limb.

### Detection of CII-protein expression on MHCII

To investigate the presentation of CII-peptide on MHCII molecules, T cell hybridomas specific for modified CII-peptides were used. Hcq3 hybridoma (recognizes glycosylated CII aa259-270), Hcq4 (recognizes naked CII aa259-270) and HdBr1 (recognizes hydroxylated CII aa259-270). To ensure proper specificity of the hybridoma, 5x10^4^ cells from each hybridoma were co-cultured for 24h with 5x10^5^ naïve unsorted splenocytes and stimulated with 100 ul of naked CII-peptide, hydroxylated CII-peptide or glycosylated CII-peptide. Analysis of IL-2 levels in supernatants was performed using sandwich ELISA (R&D Systems) and showed specific peptide responses for each hybridoma (Fig 1D). The specific T cells hybridoma were then used to investigate CII presentation on cells from peritoneal cavity, bone marrow and spleen, 12 weeks after transplantation. Peritoneal macrophages (PEM) and bone marrow cells from femur, tibia as well as splenocytes were harvested from unimmunized transplanted mice (n = 6/group) and seeded in 96-well plates in triplicates at a concentration of 5x10^5^ cells/well with either of the T cell hydridomas in a volume of 50 ul at a concentration of 5x10^4^ cells/well. To study the expression of the CII-peptide on B cells, splenocytes from unimmunized LNT-Ctrl (n = 3) and LNT-CII mice (n = 3) were depleted from T cells using a pan T cell depletion kit (Dynal, Invitrogen) and further positively selected for CD19 positive B cells (Stem Cell Technology). The selected CD19 negative and CD19 positive cell populations were seeded in a 96-well plate in triplicates at a concentration of 5x10^5^ cells/well together with 5x10^4^ Hcq4 hybridoma T cells. After 24h the levels of IL-2 in culture supernatants was measured by ELISA (R&D Systems).

### Histological examination of inflamed joints

Histopathologic examination of the joints was performed after routine fixation, decalcification, and paraffin embedding. Tissue sections from fore and hind paws were cut and stained with hematoxylin–eosin. All the slides were coded and evaluated by two blinded observers. The specimens were evaluated with regard to synovial hypertrophy, pannus formation, and cartilage/bone destruction. The degree of synovitis and destruction in every joint (finger/toes, wrists/ankles, elbows, and knees) was assigned a score from 0 to 3. Occasionally one joint was missing in the histological sections, or embedded in such a way that it was impossible to evaluate the degree of synovitis and bone/cartilage destruction. Therefore, the total score per mouse was divided by the number of joints evaluated.

### CII-specific antibodies in serum

Levels of CII-specific IgG in serum, taken at indicated time points after CII immunization, were determined by ELISA. Low binding plates (NUNC, Fisher Scientific, Gothenburg, Sweden) were coated with rat CII (1 μg/ml). Serum samples were diluted (1/6000, 1/18 000, 1/54 000 and 1/162 000) and after incubation, CII-specific IgG was detected by using biotinylated rat anti-mouse IgG, IgG1, IgG2a or IgG2b at 0.5 μg/ml (Serotec, Oxford, UK). The assay was developed using extravidin-horseradish peroxidase (HRP) and tetramethylbenzidine substrate. The reactions were stopped with H_2_SO_4_ and read in Spectra Max 340PC (Molecular Devices) at 450 nm and correction at 650 nm.

### Flow cytometry of B and T cells

B and T cells were analyzed by flow cytometry (FACS) for expression of activation and differentiation markers. Briefly, single-cell suspensions were prepared from draining lymph nodes and spleen. Cells were resuspended in PBS with 10% FCS, counted (Nucleocounter) and seeded in 96-well plates. T cells were Fc blocked (clone 2.4G2 BD) before application of surface antibodies CD4 (V450 clone RM4-5, BD), CD4 (PB clone RM4-5, BD), CD25 (APC clone 3C7, BD) or CD25 (FITC, 3C7 BD) for 20 min in room temperature (RT). B cells were stained without prior Fc blocking using v450-labeled B220 (BD Biosciences) and APC-labeled CD93 for 20 min in RT. Intracellular staining was performed using FoxP3 / Transcription Factor Staining Buffer set (eBioscience) and FoxP3 (PE clone NRRF-30 eBioscience), FoxP3 (FITC, clone FJK-16s, eBioscience) or Helios (APC Alexa Fluor 647 clone 22F6 eBioscience) for 30 min in +8C°. Isotype controls were used as negative controls. Analysis was performed by FlowJo Software (Tree Star Inc., Ashland, OR, USA) and gates for surface staining were set according to flourochrome minus one [39].

### Adoptive transfer

**Fig 3:**
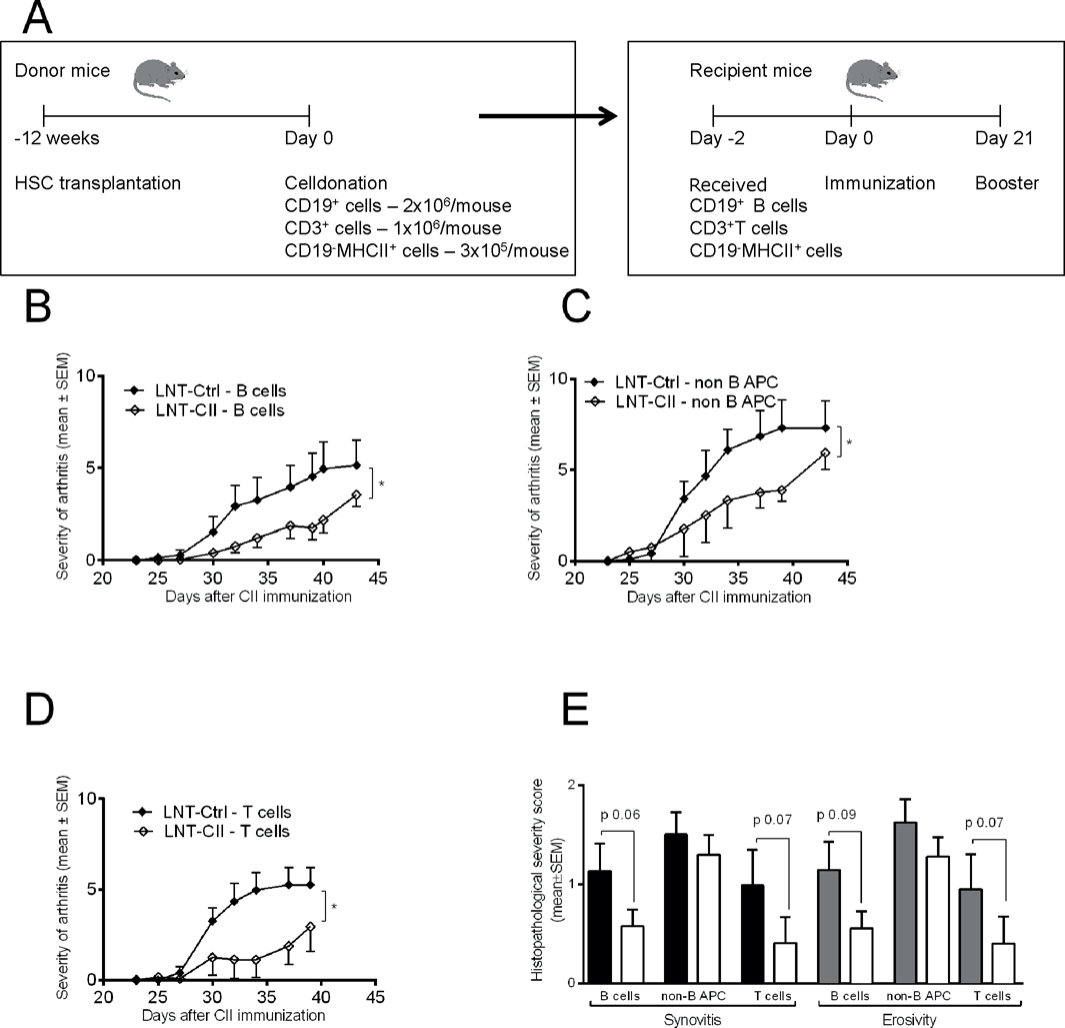
Adoptive transfer of T cells, B cells and APCs. **(A)** Design of experiment. Adoptive transfer of sorted T cells, B cells and APCs from LNT-Ctrl (n = 5) and LNT-CII (n = 6) mice respectively were transferred into naive recipients 2 days before CII immunization. **(B)** Severity of arthritis after immunization in mice receiving T cells (LNT-Ctrl n = 6, LNT-CII n = 6). **(C)** B cells (LNT-Ctrl n = 7, LNT-CII n = 7) or **(D)** APCs (LNT-Ctrl n = 6, LNT-CII n = 4). **(E)** Histopathological examination of synovitis and bone/cartilage erosivity. The experiments have been performed once. Statistical analysis was performed using linear regression for comparing development of arthritis, Mann-Whitney U-test for comparing histopathological score, mean±SEM.

Fourteen weeks after transplantation of lentivirally transduced HSCs, splenic T cells (defined as CD3^+^ cells), B cells (defined as both CD19^+^ and MHCII^+^) or the remaining MHC Class II^+^ pool (defined as CD19^-^ and MHCII^+^ cells, i.e. non-B APCs) were sorted by FACS Synergy. Briefly, single-cell suspensions were prepared in PBS with 10% FCS. APC Anti-Mouse CD3e (eBioscience), APC-Cy7 Rat Anti-Mouse CD19 (BD Pharmingen, US) and eFlour450 Anti-mouse MHC Class II (I-A/I-E) (eBioscience), respectively, were added to the cell suspensions. Following 30 min incubation at 4°C, cells were sorted by FACS. 7AA-D (BD Pharmingen, US) were added just prior sorting for exclusion of dead cells. Purity and viability of isolated cells was around 99 (S1 Fig). Freshly isolated cell populations (1x10^6^ T cells, 2x10^6^ B cells and 3x10^5^ APC cells/mouse) were adoptively transferred into naïve syngeneic recipient mice (Fig 3B–3D). Recipient mice were administered the cells 2 days prior to CII immunization and evaluated for development of arthritis as described above.

### Suppression and proliferation assays

Cells from spleen were prepared as single cell suspensions. Antigen presenting cells were purified from spleen from LNT-Ctrl mice by negative selection using Mouse Dynabeads PanT kit (Life Technologies Ltd, Paisley, UK). CD4^+^ T cells were enriched using mouse CD4 negative selection kit (Life Technologies Ltd, Paisley, UK). Cell suspension was then stained for surface expression of CD4 (FITC, clone RM4-4, BD) and CD25 (APC clone 3C7, BD) as described earlier. T cells were sorted into a purified CD4^+^CD25^-^ T effector cell population and a CD4^+^CD25^+^ T population using FACS Sy3200 Sorter (Sony Biotechnology, Champaign, USA). Cells were primarily gated as lymphocytes based on size using FSC and SSC, and to ensure that cells were singlets, cells were gated as a single cell population using FSC-A and FSC-H. Cells were then gated as CD4^+^ and then CD25^+^ for sorting. Post sorting cells were analyzed for viability using 7-AAD (BD Biosciences) and purity. Sorted APCs (50000), T effector (18000) and T_regs_ (according to ratio) cells were seeded in DMEM (supplemented with 10% FCS, 1% 2-mercaptoethanol, 1% penicillin/streptomycin, here after called complete DMEM) with added anti-CD3 (azide free, BD) at a final concentration of 0.5 μg/ml in a 96-well plate. All co-cultures were incubated for 62h at 37°C, followed by addition of 1 μl H^3^ thymidine (Perkin Elmer, Waltham, Massachusetts, USA) per well. After 12h cells were harvested and proliferation rate analyzed in Betacounter (1450 LSC & Luminescence Counter, MicroBeta TriLux, PerkinElmer, Inc, Bath, UK). Antigen-specific proliferation of whole spleen was performed at at days 14 and 28 after CII immunization. Single cells were plated in triplicates at a concentration of 5x10^5^ /well in a 96-well plate in DMEM (supplemented with 10% FCS, 1% 2-mercaptoethanol, 1% penicillinum/streptomycin) and stimulated with rat CII 50 μg/ml for 72h. As a read out, IFN-γ was analyzed in the supernatant using a DuoSet kit (R&D).

### Cell ELISA

Single cells from spleen cells and draining lymph node were plated in complete DMEM at a concentration of 5x10^5^ cells/well in a 96-well plate and stimulated with 50 μg/ml CII and standard culture conditions. The supernatant was harvested after 72h, frozen in -20°C degrees and analyzed for TNF, IL-6, IL-10, TGF-β and IFN-γ using DuoSet ELISA (R&D).

### qPCR array analysis

mRNA was prepared from lymph nodes from naïve mice (n = 3) and at from LNT-CII (n = 15) and LNT-Ctrl mice (n = 19) respectively at days 0, 3, 5, 14 and 28 after CII immunization using RNEasy Mini Kit and QIAcube (QIAGEN) with protocol RNeasy Mini Kit Cells and Tissue with DNase Digest according to the manufacturers instruction. RNA concentration was measured using a ND-1000 Spectrophotometer (NanoDrop). cDNA synthesis was made using High Capacity cDNA Reverse Transcription Kit (Applied Biosystems) including RNase inhibitor in a Veriti 96-Well Thermal Cycler (Applied Biosystems). cDNA was run in the 384-well microfluid card Taqman Array Mouse Immune Panel (Applied Biosystems/Life Technologies) detecting 90 + 6 genes using a ViiA 7 Real-Time PCR System (Applied Biosystems/Life Technologies). β-actin (*Actb*) and GADPH (*Gadph*) were selected as housekeeping genes. Relative quantification (RQ) was calculated from the sample from a naïve DBA/1 mouse.

### Statistical analysis

Statistical analyses were performed using GraphPad Prism (La Jolla, CA). Statistical differences between independent groups were calculated using the nonparametric Mann-Whitney U test for quantitative data and Fisher’s exact test for nominal data. Logistic regression was used to compare the development of arthritis between groups and linear regression was used to compare development of severity of arthritis between treatment groups. Two way ANOVA was used to compare cell proliferation in co-cultures of T cells in different ratios. Multivariate factor analysis (SIMCA-P+ software; Umetrics, Umeå, Sweden) was used to examine the relation between LNT-Ctrl and LNT-CII mice (Y-variables) and the determined gene expression levels in the qPCR array analysis (X-variables). Orthogonal projection to latent structures DAs were implemented to examine whether tolerized mice compared with non-tolerized mice could be discriminated based on the various X-variables examined. The quality was assessed based on the parameters R2, that is, how well the variation of the variables is explained by the model, and Q2, that is, how well a variable can be predicted by the model. OPLS plots in the results are based on X-variables with variable influence on projection (VIP) values >1.3 VIP values can be used to discriminate between important and unimportant predictors for the model. In the OPLS analyses, the importance of each X-variable to Y is represented by column bars. Jackknifing was used to calculate SEs displayed as an error bar on each column (representing the 95% confidence interval). Univariate analyzes by student’s t-test were performed exclusively on the X-variables *Socs1*, *IL-10*. *IFNg and IL-6* to avoid mass significance. P < 0.05 = * was considered significant. P< 0.005 **, P<*** 0.001.

## Results

### The CII-peptide is expressed in its naked form on MHCII in LNT-CII mice

To generate a tolerogenic lentiviral vector where the immunodominant CII-peptide, i.e. amino acids of CII (259–270), is presented on MHCII Aq by APCs, we cloned the CII-peptide aa259-270 DNA sequence into the CLIP position of the invariant chain (LNT-CII). The native CLIP peptide was kept as in the control vector (LNT-Ctrl) (Fig 1A) [37]. To express the CII-peptide on APCs, hematopoietic stem cells (HSC) were transduced with lentiviral particles and injected into lethally irradiated recipient mice (Fig 1B). The mice were allowed to reconstitute their hematopoietic cell populations for at least 10 weeks before CIA induction. After reconstitution we determined the expression of the CII-peptide/Aq on APCs using CII specific T cell hybridomas recognizing post-translational modified forms of the peptide [40]. All hybridomas were functional and responded as expected to their respective peptide (Fig 1C), and we found that it was predominantly the naked non-modified variant CII-peptide that was expressed in all assessed tissues: bone marrow, spleen and on peritoneal macrophages both before (Fig 1D) and day 28 after CII-immunization (S1 Fig). In spleens depleted of T cells both CD19 positive B cells as well as CD19 negative cells also expressed the naked non-modified variant of the CII-peptide (Fig 1E and S1 Fig).

### Frequency and severity of arthritis are ameliorated in LNT-CII mice

The LNT-CII mice showed an almost complete resistance to develop arthritis (severity and frequency), by contrast to the LNT-Ctrl mice that developed a rapidly progressing arthritis (Fig 2A and 2B). These findings were confirmed by histopathology of the joints, which showed that only 6% of LNT-CII mice had mild signs of synovitis and erosivity, whereas >90% of LNT-Ctrl mice developed severe synovitis, and cartilage and bone destruction (Fig 2C and 2D). Thus, lentiviral gene therapy using LNT-CII that presents naked CII-tolerogen on APCs prevents development of CIA.

### CII-specific IgG B cell responses are down-regulated in LNT-CII mice

It is known that the B cells and their production of CII-specific antibodies are crucial for the development of CIA [41,42]. The serum levels of CII-specific IgG antibodies, including the subclasses IgG1, IgG2a and IgG2b, were significantly lower in LNT-CII compared to LNT-Ctrl mice throughout the experiment (Fig 2E and 2F). This response was not due to a general effect on total immunoglobulin levels, which were unchanged (S2 Fig). The CII-specific IgM response was similar in both groups of mice both before and at days 29 and 47 after CII immunization (S2 Fig). These findings indicate that only the T cell dependent production of antigen-specific IgG is affected by the induced tolerance.

### B cells and non-B cells mediate tolerance to arthritis in LNT-CII mice

There are three main cell types that can contribute to immunological tolerance: Treg, APCs and B cells. To investigate the role of each of these cell population in our tolerance model, we isolated splenic T cells, B cells and the remaining non-B cell APC pool from mice after gene therapy (S1 Fig). The isolated cell populations were transferred into naïve syngeneic recipients two days before CIA was induced (Fig 3A). There were no differences between the two groups of mice in the composition and proportion of cells before transfer with respect to leukocytes, lymphocytes, CD4^+^ T cells, CD4^+^ Foxp3^+^ Tregs, MHCII^+^CD19^+^ B cells or MHCII^+^CD19^-^ non-B cell APCs (S3 Fig and Table 1). The macroscopically observed development of arthritis was significantly ameliorated after transfer of splenic B cells (Fig 3B), non-B APCs (Fig 3C) or T cell from LNT-CII mice (Fig 3D). However, histopathological examination showed that transfer of B cells, but also of T cells from LNT-CII mice reduced the degree of synovitis and cartilage and bone destruction (Fig 3E). The histopathological differences in the joints between the groups after transfer of non-B cell APCs were not as pronounced as for the other cell types transferred, though this could be due to the lower amount of cells transferred (Fig 3E). At the termination of the T cells transfer experiment we analyzed the serum levels of CII-specific IgG in total and of all subclasses and found no statistical differences between the respective groups (S4 Fig). Thus, transfer of T cells, B cells and non-B cell APCs from LNT-CII mice suppressed the macroscopically observed severity of CIA, which indicates that tolerance can be induced and maintained by multiple cell types.

**Table 1:** Proportion of CD4^+^Foxp3^+^ Tregs of CD4^+^ cells in spleen and draining lymph nodes before and after immunization.

Tissue	Mice	Day				
		0	3	5	28	49
**Spleen**	**LNT-Ctrl**	26.1 ± 0.6	22.6 ± 0.6	25.9 ± 0.2	28.0 ± 1.2	21.5 ± 1.6
**Spleen**	**LNT-CII**	25.5 ± 1.8	26.1 ± 0.2**	24.2 ± 0.3*	24.1 ± 1.0*	21.1 ± 1.2
**Lymph nodes**	**LNT-Ctrl**	11.8 ± 0.9	12.0 ± 0.8	12.6 ± 0.7	ND	ND
**Lymph nodes**	**LNT-CII**	12.37 ± 0.4	14.9 ± 0.6*	11.7 ± 0.9	ND	ND

*p<0.05 and

**p<0.01 LNT-Ctrl vs LNT-CII. Data presented as mean ± SEM. At day 0, 3, 5 and 28 n = 3, at day 49 n = 5–6 per group. ND; not done.

### The suppressive Treg function is enhanced in LNT-CII mice

**Fig 4:**
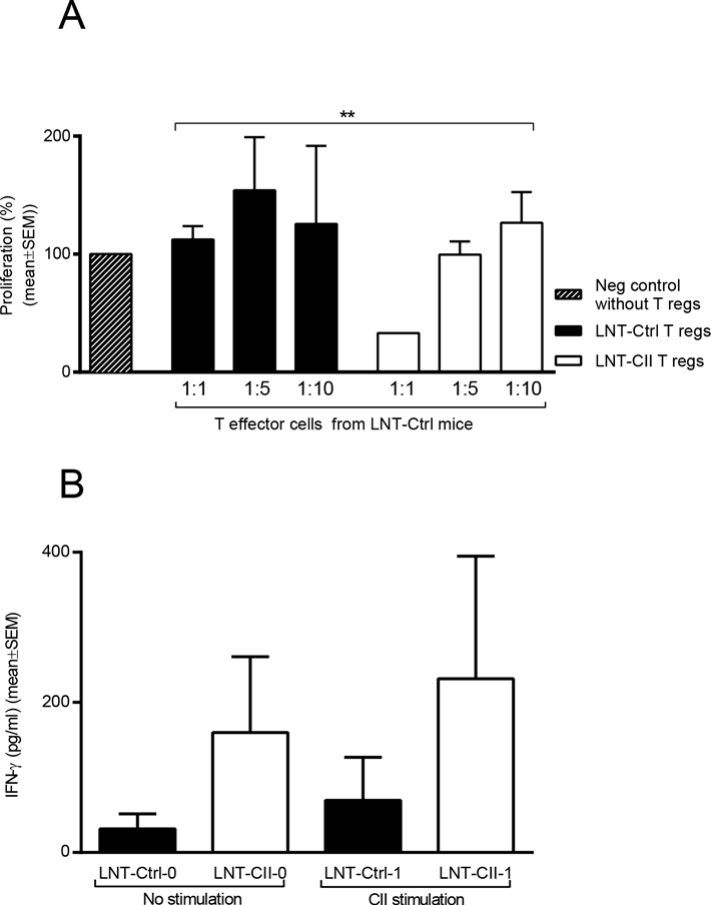
Involvement of T cells in tolerance induction. **(A)** Capacity of Tregs cells to down-regulate effector T cell responses. Sorted Tregs cells from LNT-Ctrl or LNT-CII mice were co-cultured with LNT-Ctrl T effector cells in ratios 1:1, 1:5 and 1:10 for three days and proliferation measured by radioactive thymidine incorporation (counts per minutes). Y-axis—percentage of proliferation compared to the negative control containing sorted T effector cells only (n = 6/group). The experiment has been repeated three times. **(B)** CII-specific IFN-γ production from stimulated spleen cells obtained 14 days after CII immunization, n = 4–5 and repeated once. Statistical analysis was performed using Two-way ANOVA for evaluation of proliferative responses in (A) and Mann-Whitney U-test in (B), mean±SEM.

Induction of tolerance by T cells could potentially be attributed to Tregs [43–45]. We assessed the proportion of Tregs (CD4^+^Foxp3^+^ cells) at days 0, 3, 5, 28 and 49 after CIA induction. The proportion of Tregs in the spleen and lymph nodes was increased at day 3 in LNT-CII compared to LNT-Ctrl (Table 1). However, at day 5 the proportions of Tregs were reduced in LNT-CII compared to those in LNT-Ctrl mice (Table 1, gating strategy S5 Fig). This suggests that events crucial for tolerance take place very early after immunization with CII. To investigate whether the suppressive function of Tregs was enhanced in LNT-CII mice, we purified T effector cells (CD4^+^CD25^-^) from LNT-Ctrl and Tregs (CD4^+^CD25^+^) from immunized LNT-CII and LNT-Ctrl mice (S6 Fig). Stimulation with anti-CD3 antibodies showed that Tregs from LNT-CII but not LNT-Ctrl mice significantly reduced the proliferative response of the T effector cells (Fig 4A). Unexpectedly, unsorted spleen cultures (characterized in S7 Fig and S8 Fig) from LNT-CII mice stimulated with CII showed a tendency to increased IFN-γ production at day 14 compared to LNT-Ctrl mice (Fig 4B). Taken together, this suggests that Tregs from LNT-CII mice are an important mediator of the tolerogenic phenotype.

### LNT-CII has limited effect on cytokine levels

Pathogenesis of CIA is characterized by a Th1 and Th17 response that is counter-acted by regulatory cytokines such as IL-10, IL-4 and TGF-β [46,47]. To analyze the cytokine pattern during the course of CIA mRNA levels of TNF-α, IFN-γ, IL-4 and IL-17A in draining lymph nodes were determined. In addition, protein levels of TNF-α, IFN-γ, IL-6, IL-10 and TGF-β in serum as well as draining lymph node cultures at day 49 after immunization were assessed. Despite the significant differences in arthritis outcome, there were no differences between the groups in the levels of any of these mRNA or proteins except IFN-γ, which was decreased in lymph node cultures from LNT-CII (mean ± SEM pg /ml; 22.1 ± 3.86) compared to LNT-Ctrl (50.08 ± 8.8, p = 0.03).

### Early increase in *Socs1* expression levels is associated with tolerance

**Fig 5:**
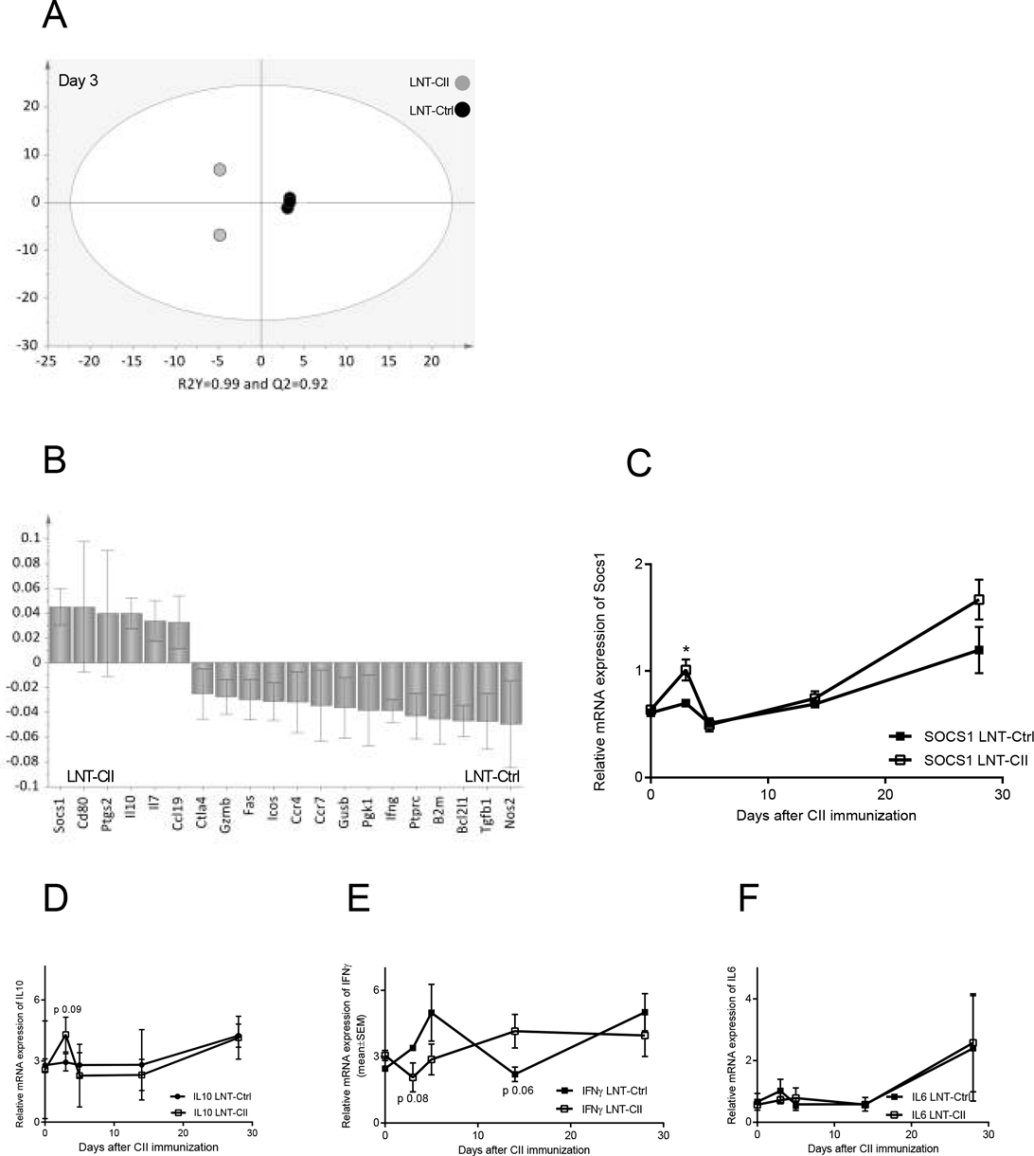
qPCR array and SOCS1 association with LNT-Ctrl vs LNT-CII at day 3 after CII immunization. **(A)** OPLS-DA scatter dot plot showing the separation of gene expression in tolerized or non-tolerized mice and **(B)** OPLS-DA column loading plot that depicts the association between LNT-CII and LNT-Ctrl mice with the expression of different genes. X-variables represented with a positive bar are positively associated with LNT-CII mice, whereas variables in the opposite direction are inversely related to this group of mice. The larger the bar and smaller the error bar, the stronger and more certain is the contribution to the model. The final OPLS-DA loading plots are based on parameters with VIP values ≥1.3. R2Y indicates how well the variation of Y is explained, whereas Q2 indicates how well Y can be predicted. Univariate analysis (Student’s t-test) of **(C)** SOCS1 **(D)** of IL-10 **(E)** IFN-γ **(F)** IL-6, n = 2–4 animals/group, mean ± SEM. *Actb* and *Gadph* were selected as housekeeping genes and relative quantification was calculated from the sample of a naïve DBA/1 mouse.

As it proved difficult to explain the tolerant phenotype by one single factor at a certain time point, an immune card array that detects mRNA levels of 90 different genes was employed on draining lymph nodes 0, 3, 5, 7, 14 and 28 days after CII immunization. Multivariate discriminant analysis, OPLS-DA, indicates that the gene expression profile is different in LNT-CII *vs* LNT-Ctrl mice at all time points (Fig 5A and S9 Fig). The variables that displayed the strongest association (positive or negative) with either group of mice are identified in the column plots (Fig 5B and S9 Fig). Variables with a positive bar are positively associated with LNT-CII mice whereas variables with bars in the opposite direction are inversely related to the LNT-CII mice.

At day 3 after CII immunization the OPLS-DA analysis showed that the mRNA expression of *Socs1* along with *Il10* associated strongly with LNT-CII mice, while *Ifng* associated with LNT-Ctrl mice (Fig 5B). The individual values of mRNA expression of *Socs1*, *Il10*, *Ifng and Il6* during the course of arthritis are plotted in Fig 5C–5F. Day 3 after CII immunization is also the only time point where the proportion of Tregs was increased in tolerized LNT-CII mice (Table 1). Thus, the early increase in *Socs1* mRNA coincides with the increased frequency of Tregs and resistance to arthritis development.

## Discussion

In this study we demonstrate that transfer of hematopoietic stem cells expressing the CII-peptide on MHCII Aq (LNT-CII) to lethally irradiated mice induces an almost complete antigen-specific tolerance CIA. We show that only 5% of the CII-tolerized mice developed a mild synovitis while more than 95% of control mice developed a severe synovitis as well as severe cartilage and bone destruction. We found that early events including up-regulation of SOCS1 and an increased proportion of Tregs associated with resistance to arthritis development. We demonstrate that the tolerance to CIA caused by excessive presentation of the CII-tolerogen on APCs is a process with multiple immunological effector mechanisms, as transfer of both T cells, B cells and to some extent also MHCII^+^ cells from CII-tolerized mice to naïve recipients ameliorate the course and severity of arthritis. Further, the OPLS-DA analyses of mRNA expression from peripheral lymph nodes clearly shows that tolerance mechanisms differ substantially between CII-tolerized and control mice but also between different time points throughout the course of CIA.

It is well known that Tregs are generated both in the thymus and in peripheral tissues [48] and that functional Tregs cells are pivotal to maintain immunological homeostasis [49]. We demonstrate that early after CII immunization (day 3) there is an increased proportion of Tregs and up-regulation of the mRNA expression of SOCS1 and IL-10 in tolerant LNT-CII mice. At the same time, LNT-CII mice also down-regulate mRNA expression of IFN-γ and to some extent IL-6. IL-10 is a known inducer of SOCS1 [50], and we have previously shown that the CIA is alleviated in the presence of an increased IL-10 production, which coincided with an increased SOCS1 expression in draining lymph nodes and reduced serum levels of IL-6 [34]. SOCS1 prevents JAK/STAT signaling, and plays an important role in Tregs by maintaining Foxp3 expression and by suppressing IFN-γ and IL-17 production [51]. Thus, we hypothesize that tolerance is established very early after CII immunization, and that it involves both generation of Tregs as well as SOCS1 that shuts down the CII-specific effector response. This hypothesis is further supported by clinical studies showing that tolerance induction is more successful if the host’s immune system has a tolerogenic phenotype [52].

We also found that suppressive capacity of Tregs from tolerant LNT-CII mice was preserved after general stimulation with anti-CD3, while the suppressive capacity of LNT-Ctrl Tregs was decreased. This may be due to the inflammatory status in these animals, since it has been shown that in RA the Tregs are under the influence of the cytokine milieu and that this has a negative effect on their suppressive function, e.g. anti-TNF treatment has been shown to improve Treg function [53]. Another explanation could be that the proportion of antigen (CII)-specific Tregs and T effector cells in LNT-CII is altered compared to LNT-Ctrl mice. It is likely that both an increased induction of antigen (CII)-specific Tregs as well as fewer antigen (CII)-specific T effector cells operate in LNT-CII mice as the CII-peptide is presented on both B cells and non-B cell APCs.

Earlier studies have highlighted that post-translational glycosylation of the CII-epitope is important for arthritis development in the CIA model [54]. Further, CII-autoreactive T cells that recognize the glycosylated CII-epitope have been found in blood and synovial fluids from patients with RA, and there is a significant correlation between disease activity and T cell response to the glycosylated CII-epitope [55]. It has also previously been shown that overexpression of the glycosylated CII-epitope is superior to the naked epitope in tolerance induction to CIA [14]. We show that it is the naked rather than the glycosylated CII-peptide that is presented, which can be due to difficulties for the APC to glycosylated the CII-peptide in our experimental system. Nevertheless, overexpression of the naked CII-peptide induces tolerance to CIA [37,38], though it is possible that the tolerogenic effect might have been even better if the glycosylated CII-peptide was expressed. The discrepancies between the findings regarding the tolerogenic effects of glycosylated and naked CII-peptide may be explained by yet unpublished work showing that it is the non-modified naked CII peptide that is presented in thymus (personal communication, J Bäckström, KI). This implies that central tolerance mechanisms are important and it would in future studies be very interesting to perform thymectomy prior to irradiation, repopulation and induction of arthritis to further decipher the puzzle of tolerance. Also supporting a role for central tolerance in autoimmune arthritis is the fact that aberrant ZAP-70, a key signal transduction molecule in T cells during thymic selection changes the thresholds of T cells to thymic selection, leading to the positive selection of otherwise negatively selected autoimmune T cells and to subsequent development of chronic autoimmune arthritis in mice [56].

Intravenous administration of lentiviral particles encoding LNT-CII/LNT-Ctrl either before or after CII immunization induces partial tolerance to CIA [37,38], while transplantation of lentivirally transduced HSCs induces an almost complete tolerance. The main reasons in efficacy between the two delivery systems are most likely the amount of time between transfer of lentiviral particles and CII immunization, and the impact as well as the amount of the CII-peptide expression in the mouse. After transfer of transduced HSCs, the immune system was allowed to repopulated for > 10 weeks, while the lentiviral particles were injected either 30 days before or, at different time points after CII-immunization. Transfer of transduced HSCs to a lethally irradiated mouse leads to differences in function, but not in proportions, of immune cells already before CII-immunization (Fig 5 and S9 Fig). It is also well known that presentation of a high amount of antigen without co-simulation leads to tolerance. The expression of the CII-peptide was detectable with the T cell hybridomas after transfer of lentiviral transduced HSCs (Fig 1D), but not after intravenous injection that indicates that there is a lower presentation of the peptide.

B cells are potent professional APCs that have been previously implicated in tolerance induction and disease development [27,57,58]. The B cells contribute to CIA both by presenting antigens and by producing cytokines and CII-specific antibodies [59]. In the tolerogenic LNT-CII mice, there is a profound reduction of the B cell response, and as this require T cell activation, these data support an increased suppression by Tregs and a reduction in antigen-specific T effector cells. Further, self-antigen expression by B cells has been shown to be very efficient in inducing Tregs in the periphery, whereas self-antigen expression by DC led mainly to deletion and anergy of antigen-specific T effector cells [24].

From day 5 and onwards after CII immunization we could not detect a general increase in the proportion of Tregs in the tolerant LNT-CII mice, but rather the opposite as the proportion of Tregs increased in the arthritic LNT-Ctrl mice. This could be explained by the absence of an ongoing immune response in LNT-CII mice, while the arthritic LNT-Ctrl mice need to mobilize their Tregs to limit the ongoing inflammation, as it is well known that Tregs increase during inflammation [60].

The kinetics of IFN-γ in the LNT-CII mice is interesting. The levels are decreased compared to LNT-Ctrl mice at day 3, increased at day 14, similar at day 28 and decreased at day 49 after CII immunization. This most likely mirrors that there are different effector mechanisms governing the tolerogenic state at different time points. IFN-γ has both regulatory and immune stimulating properties. It can inhibit osteoclastogenesis and formation Th17 cells, as well as facilitate the induction and increase the suppressive capacity of Tregs, and in addition up regulate MHCII as well CD80/86 expression (reviewed in [61]). IFN-γ is also of importance for B cell responses and IFN-γ production by T follicular helper cells is necessary for germinal centre formation and the production of high affinity antibodies [62].

Regulatory B cells can also influence the inflammatory response [63]. Regulatory B cells are defined either by IL-10 production or by surface markers including for B220/CD23/CD24/IgM/CD1d [58], however our data (not shown) do not support an increased B regulatory cell compartment. Thus, the reduced B cell response in tolerant LNT-CII mice is most likely secondary to lack of a CII-specific T cell response.

To summarize, we have shown that a sustainable tolerance against CIA can be established by endogenous expression of naked unglycosylated CII-peptide on MCHII. Tolerance is established very early during the course of arthritis and can be mediated by both B and non-B cells as antigen presenting cells. We suggest that the model we have established using lentiviral constructs can be used to elucidate the detailed functions of not only tolerogenic states in CIA, but also in other experimental autoimmune models.

## Supporting Information

S1 Fig Gating strategy, purity and viability of cells used in CII-peptide presentation assay and adoptive transfer and CII-peptide expression. **(A)** Detection of the CII-peptide in splenocytes by T cell specific hybridomas at day 28 after transfer of lentivirally transduced HSCs. **(B)** Verification of the functionality of the hybridomas. **(C)** Purity of non-B cells APCs and B cells used for the experiment in Fig 1E. **(D)** FACS sorting of B cells and T cells was initiated through gating for BSC area vs FALS area followed by gating for 7-AAD. T cell sorting was performed by gating for CD3^+^ or CD19^-^ cells. Sorting of B cells was performed gating for MHCII positive CD19^+^ cells and sorting non B cell APCs gating for MHCII^+^ CD19^-^ cells. **(E)** Flow cytometry using BSC area (y-axis) vs 7-AAD (x-axis) to estimate cell viability and purity of the sorted cells using MHCII (y-axis) vs CD19 (x-axis) for B cells and CD3 (y-axis) vs CD19 (x-axis) for T cells.
(EPS)

S2 Fig Serum levels of general IgG and CII-specific IgM. **(A)** Serum levels of general IgG at day 49 after CII immunization, n = 7+6 mice. **(B)** Serum levels of CII-specific IgM antibodies at day 0, 27 and 49 after CII immunization, n = 6+6 mice.Goat anti-mouse polyclonal IgG antibodies (Jackson Immunology Research, Suffolk, England) was used as coating, and 2% BSA (Sigma-Aldrich) for blocking. Serum samples were serially diluted from 1/ 7500 to 1/202 500) The total IgG levels in serum was detected by a biotinylated goat anti-mouse IgG (Southern Biotechnology, Alabama, USA) or biotinylated (Fab)_2_ goat antimouse IgM (Jackson ImmunoResearch Laboratories). The assays were developed using extravidin-horseradish peroxidase (HRP) and tetramethylbenzidine substrate. The reactions were stopped with H_2_SO_4_ and read in Spectra Max 340PC (Molecular Devices) at 450 nm and correction at 650 nm. Data were expressed as optical density (OD).
(EPS)

S3 Fig Cell population before and after CII immunization. **(A)** The absolute number of leukocytes and lymphocytes in blood before CII immunization, n = 6+7 mice were counted in a Sysmex Cell counter. The distribution of **(B)** CD4^+^, CD19^+^MHC II^+^ and CD19^-^MHC II^+^ cells in blood before CII immunization, **(C)** lymph nodes and **(D)** bone marrow. **(E)** Intracellular expression of Foxp3 and CTLA in CD4^+^CD25^+^ T cells from lymph nodes before CII immunization, n = 3+4 mice. **(F)** Expression level (MFI) of CD62L on CD4^+^ cells in blood **(G)** MFI of MHCII on CD19^+^ and **(H)** CD19^-^ cells in blood before and during the course of arthritis, each mouse is shown as individual dots. The cells were stained for flow cytometry as previously described.
(EPS)

S4 Fig Serum levels of CII-specific IgG after adoptive transfer of T cells, day 39 after CII immunization. The different subclasses of IgG as well of CII-specific total IgG are indicated, n = 6+6 mice.
(EPS)

S5 Fig Gating strategies and phenotype of Tregs. (EPS)

S6 Fig Phenotypes of cells in the T cell suppression experiments. **(A-B)** Gating strategy and purity of CD4^+^CD25^+^ T cells in the T cell suppression assay (Fig 4A). **(C)** Purity of T cell depleted antigen presenting splenocytes used in the T cell suppression assay (Fig 4A).
(EPS)

S7 Fig Phenotype of B cells and non-B cell APC at day 14 after CII-immunization. The following antibodies for flow cytometry CD21-Fitc, CD23-PE-Cy7, CD93-APC, CD19-V450, IgD-bio/PerCP and MHCII-PE were used.
(EPS)

S8 Fig Phenotype of CD4 positive T cells in spleen at days 14 and 28 after CII-immunization. (EPS)

S9 Fig qPCR array and SOCS1 association with LNT-Ctrl vs LNT-CII at days 0, 5, 14 and 28 after CII immunization. **(A, C, E, G)** OPLS-DA scatter dot plot showing the separation of gene expression in tolerized or non-tolerized mice. **(B, D, F, H)** show the OPLS-DA column loading plot that depicts the association between LNT-CII and LNT-Ctrl mice with the expression of different genes. X-variables represented with a positive bar are positively associated with LNT-CII mice, whereas variables in the opposite direction are inversely related to this group of mice. The OPLS-DA column plots are based on variables with VIP values ≥1.3. R2Y indicates how well the variation of Y is explained, whereas Q2 indicates how well Y can be predicted.
(EPS)
